# Comment on Aasebø, E., et al. “The Progression of Acute Myeloid Leukemia from First Diagnosis to Chemoresistant Relapse: A Comparison of Proteomic and Phosphoproteomic Profiles”. *Cancers* 2020, *12*, 1466

**DOI:** 10.3390/cancers12092461

**Published:** 2020-08-31

**Authors:** Peter DeRosa, Victor Nava

**Affiliations:** 1 Veterans Affairs Medical Center, Washington, DC 20422, USA; Victor.Nava@va.gov; 2 Department of Pathology, George Washington University, Washington, DC 20037, USA

In a recent article published in this journal, Aasebø and colleagues reported [1] increased levels of various proteins, including CD36, in acute myeloid leukemia (AML) cells at relapse, when compared with the time of first presentation, and suggest targeting these proteins for direct therapeutic strategies to alleviate chemoresistance in relapsed AML. Interestingly, CD36 is a scavenger receptor that mediates lipid uptake, and is gaining attention in clinical trials as a potential druggable target in cancer treatment [2]. Using next generation sequencing (NGS) in a bone marrow specimen from a patient with AML and synchronous plasma cell myeloma, we have identified a CD36/Y325* mutation, which would encode a truncated protein lacking the signaling intracytoplasmic carboxyl-terminus end. This specific mutation is not retrievable in PubMed or database searches (ClinVar, HGMD, COSMIC and others).

However, a detailed literature search revealed that only one prior publication described the same mutation in AML [3]. Therefore, we speculate that this specific mutation may confer a survival advantage to neoplastic cells, since wild type CD36 has been shown to be involved in apoptosis by activation of caspase 3 [4]. In summary, we have independently identified an unrecognized CD36/Y235* mutation in a case of AML by NGS, which underscores the strength of this technology to decipher genetic alterations and further supports a possible oncogenic role of CD36 [2].
